# Clinical Lectures on Diseases of the Eye

**Published:** 1866-03

**Authors:** E. L. Holmes

**Affiliations:** Lecturer on Diseases of the Eye in Rush Medical College, and Surgeon to the Chicago Charitable Eye and Ear Infirmary


*Clinical Lectures on Diseases of the Eye.* By E. L. Holmes, M. D., Lecturer on Diseases of the Eye in Rush Medical College, and Surgeon to the Chicago Charitable Eye and Ear Infirmary.

HYPERMETROPIA—PRESBYOPIA.

Gentlemen,—The class of cases, which now in order claims our consideration, is interesting as having till within a few years been almost wholly overlooked by the most careful observers. The credit of first analyzing these cases has often been given to Donders; priority is now conceded, I believe, to Stellwag, of Vienna. The class is characterized by such a condition of the refracting media of the eye, that *converging* rays of light may be brought to a focus on the retina, when the apparatus of adjustment is in a state of relaxation. This condition has been called Hypermetropia, and is an important subject in the science of ophthalmology. You can better appreciate this condition of the eye, by recalling the fact, that in the normal eye, the apparatus of accommodation, in a state of full relaxation, causes parallel or very slightly diverging rays to come to a focus *on* the retina; consequently, very distant objects may be seen distinctly. In Hypermetropia, parallel rays come to a focus, when the apparatus of adjustment is in a state of relaxation, *behind* the retina. As converging rays, on passing through a convex lens, come to a focus at a point nearer the lens than the focus of parallel rays, and as the retina is at a point nearer the crystalline than the focus of parallel rays, the eye is in the case supposed adjusted for converging rays. As all objects, whether near or distant, send off only *divergent* rays, such a patient, with the ciliary muscle in a state of complete relaxation, cannot see distant objects. To do this, with the eye in this condition, he is obliged to place before the cornea a suitable double convex lens, which renders parallel rays *convergent.* We have already seen that the more convex a lens is, the less distant behind it is the point at which parallel rays are brought to a focus. Hence, a patient with Hypermetropia, to see distant objects without the aid of a convex lens, must cause the ciliary muscle to contract, and thus produce a greater degree of convexity of the crystalline lens. Such a patient, in looking at distant objects, is obliged to make a muscular effort on the part of the adjusting apparatus, while the normal eye can recognize distant objects without this effort; or, in other words, when the ciliary muscle is in a state of relaxation. As objects are brought nearer the eye, a still greater effort of the ciliary muscle is required, until finally, in endeavoring to read, for instance, the whole possible muscular power is expended. Such a patient, in using the eyes for near objects, is usually obliged to employ so much muscular effort, that a painful sense of fatigue is soon experienced. In some cases, there is a greater power in the ciliary muscle to increase the convexity of the lens than exists in the normal eye, which aids the patient in overcoming the difficulty in vision.

On the other hand, there are extreme cases in which the patients are unable to see distant objects, not only when the ciliary muscle is in a state of complete relaxation, and consequently the lens least convex, but even when the muscle is in its state of greatest convexity, from the utmost contraction of the ciliary muscle. In such an eye, the images of distant, as well as of near objects, are formed behind the retina. A patient in this condition, can see neither near nor distant objects distinctly. Such patients are in the habit of bringing objects near the eye, since the indistinctness relatively decreases with the increase in the size of the images on the retina, even though indistinct.

For these reasons, ophthalmologists were formerly misled, attributing the indistinctness of vision to an amaurotic condition of the eye.

By placing a minute aperture in a thin plate of metal as near the cornea as possible, diffused light is excluded, and objects are seen somewhat more distinctly. To produce the same effect, patients are in the habit of nearly closing the lids in the act of looking.

In typical cases of Hypermetropia, the cause of the imperfect vision depends upon an abnormal shortness of the antero-posterior diameter of the globe, which may be congenital or acquired. The retina is too near the cornea. By recalling the manner in which parallel and diverging rays of light are affected in passing through lenses of different degrees of convexity, you can readily understand how, in such cases, the images of distant objects, without an effort of the ciliary muscle, must be formed behind the retina.

Hypermetropia depends often on a undue flattening of the cornea and crystalline lens, from absorption of the fluids of the eye. The removal of the lens by accident, or in the operation for cataract, renders an eye hypermetropic. Certain diseases of the choroid, by infiltration and thickening of its substance, is said to produce Hypermetropia, by throwing the retina slightly forward towards the lens.

Hypermetropia has been observed as an apparent symptom of incipient atrophy of the optic nerve. It is often produced in an eye which has been long in disuse, as in certain cases of strabismus. From want of exercise, the ciliary muscle becomes weakened and unable to increase the convexity of the lens; the latter in turn, from constant traction of the Zonula of Zinn, becomes more and more flattened, till even parallel rays of light are brought to a focus behind the retina. On the other hand, Hypermetropia is very often the cause of strabismus. Although reasons for this last phenomenon have been suggested, a perfectly satisfactory explanation has not yet been found.

Hypermetropic patients are very liable to suffer a peculiar neuralgic pain in and around the eyes, after working even for a a short period upon near and fine objects. Such patients are often unable to pursue any occupation requiring constant use of the eyes. They soon experience a sense of fatigue, which at length becomes pain. Rest will cause this discomfort to subside; only to return, however, on resuming the customary labor. Continued efforts soon produce not only pain but a decided congestion, with a peculiar irritable condition of the eyes, which renders any occupation almost impossible.

The treatment in cases of Hypermetropia consists in the careful observance of such habits of life, especially with children, as will tend to prevent undue exercise of the eyes, and in the use of suitable double convex lenses.

In slight cases, a correct diagnosis can only be made after a careful examination. It is impossible to know without this, whether a patient, in looking at a distance, sees objects with the ciliary muscle in a state of relaxation, as in the normal eye, or whether he sees them by making an effort with the muscle of accommodation. To decide this point, it is necessary usually to paralyze the ciliary muscle by means of atropine. If a drop of a solution of sulphate of atropia, gr. iv. to the ounce, be instilled upon the conjunctiva every fifteen minutes for two or three hours, the muscle will be completely paralyzed, and the lens will assume its least possible degree of convexity. A normal eye thus influenced by atropine, would readily distinguish distant objects. But in the hypermetropic eye, as we have seen, the rays from distant objects are brought to a focus behind the retina; consequently, such objects could not be seen distinctly. By placing before the eye a suitable convex lens, the rays are rendered converging, and on thus passing through the cornea and crystalline lens, are brought to a focus, not behind the retina, but on it. After the effects of the atropine have passed away, if the same lens be placed before the eye, distant objects can be seen without any effort on the part of the apparatus of accommodation. The eye thus provided with a lens, instead of being obliged to use a certain portion of its adjusting power, will, like the normal eye, see at a distance *without* muscular effort, and can reserve its whole power of adjustment for near objects.

Occasionally we meet with patients in whom one eye is myopic, and the other hypermetropic. In the case of the clergyman, whom a few of you recently saw, the myopia of one eye and the Hypermetropia of the other eye were very marked.

To recapitulate: in the normal eye the apparatus of accommodation can adjust the focus for objects at any distance, more than five inches from the cornea; but when the eye is accommodated for very distant objects, the ciliary muscle is in a state of perfect relaxation, and the lens reduced to its least degree of convexity. To this normal condition of the eye has been given the term Emmetropia—vision in due measure.

Of the abnormal conditions of the globe, we have, 1st, Brachymetropia—shortsightedness—in which the rays of light are brought to a focus *in front* of the retina, in consequence of the abnormal distance at which the latter is placed from the lens; 2d, the opposite of the condition just mentioned, in which the retina is too near the lens, in consequence of an abnormal shortening of the diameter of the eye. To this condition has been given the term of Hypermetropia—vision beyond normal measure, "oversightedness." The apparatus of accommodation may, in each of these cases, be perfectly normal. It should be remembered that the faculty of accommodation, properly speaking, is confined to the power of increasing or diminishing the convexity of the lens, which changes cause rays of light from objects at different distances to be brought to a focus at the same instance behind the lens. If the retina is situated either too far from the lens, as in shortsightedness, or too near the lens, as in Hypermetropia, a part of the power of adjustment is expended in moving the focus, in the first case, further from the lens, and in the second, towards the lens.

There are two abnormal changes in the apparatus of accommodation, the form of the eye remaining normal: 1st, Plesiopia—nearsightedness as we have already described, in which the lens can be rendered more convex, but cannot be sufficiently flattened. 2d, The opposite of this—Presbyopia (old)—farsightedness, in which the lens can be flattened sufficiently to see distant objects distinctly, although its convexity cannot be increased sufficiently for seeing near objects. Hence, as the power of accommodation is destroyed as regards near objects, it is necessary, in looking at such objects, for presbyopic patients to increase the refraction of the rays by the use of convex lenses; and in marked cases, it is necessary to employ strong lenses for very near objects, and lenses of less convexity for objects which are moderately remote.

I am aware that I have attempted to express in a few words what requires much more space than we can now devote to this subject. If you will, however, carefully impress upon your minds a few of the important facts connected with the influence of convex and concave lenses on rays of light, and will also study the principles given in this and the past lectures, you will be able to examine and comprehend the nature of the cases which may fall under your observation.

